# Verapamil HCl demonstrates antiviral activity against porcine reproductive and respiratory syndrome virus by regulating Ca^2+^ influx and promoting type I interferon production

**DOI:** 10.1128/spectrum.03280-25

**Published:** 2026-03-05

**Authors:** Tongtong Wang, Li Chen, Jiamin Fan, Zhiqiang Yan, Wenjing Chen, Yuyu Zhang, Yulin Xu, Qinghai Ren, Shujing Wang, Siding Liu, Liangliang Li, Yubao Li, Jiaqiang Wu

**Affiliations:** 1 Key Laboratory of Livestock and Poultry Multi-omics of MARA, Institute of Animal Science and Veterinary Medicine, Shandong Academy of Agricultural Sciences 722363 , Jinan, China; 2 College of Agriculture and Biology, Liaocheng University 697434, Liaocheng, China; 3 College of Animal Science and Technology, Shandong Agricultural University 34734, Tai’an, China; Regional Centre for Biotechnology, Faridabad, Haryana, India

**Keywords:** Verapamil HCl, PRRSV inhibitor, calcium, IFN response, p38/Nrf2/Keap1**/**HO-1

## Abstract

Porcine reproductive and respiratory syndrome virus (PRRSV) poses a major challenge to swine health, impairing reproductive performance and causing respiratory disorders in pigs. Thus, it creates a considerable economic burden for the global pig production industry. Current prevention and control strategies for PRRSV remain largely ineffective, necessitating the development of novel antiviral drugs. In the current study, we examined how Verapamil HCl could potentially protect host cells against PRRSV infection using both cellular and animal models. Further, we explored the molecular mechanisms underlying these potential anti-PRRSV effects. We discovered that Verapamil HCl suppresses PRRSV infection in MARC-145 cells, PK-15^CD163^ cells, and porcine alveolar macrophages. Mechanistic analyses revealed that Verapamil HCl inhibits PRRSV-induced calcium influx in susceptible cells. Additionally, we found that Verapamil HCl enhances heme oxygenase-1 production by activating the p38/Nrf2/Keap-1/HO-1 signaling pathway, subsequently triggering antiviral interferon responses. Finally, animal experiments demonstrated that Verapamil HCl administration significantly reduces viral replication and decreases pulmonary damage in piglets. Collectively, these results suggested that Verapamil HCl may serve as a promising new therapeutic option for treating PRRSV infections.

**IMPORTANCE:**

Verapamil HCl is known to suppress infections caused by several viruses, including respiratory syncytial virus, bovine herpesvirus 1, and SARS-CoV-2. However, the potential anti-PRRSV mechanisms of Verapamil HCl remain unknown. This study demonstrates that Verapamil HCl exerts anti-PRRSV effects by attenuating the Ca^2+^ imbalance induced by PRRSV. Furthermore, Verapamil HCl can promote interferon-mediated antiviral responses and reduce pro-inflammatory factor production during PRRSV infection via p38/Nrf2/Keap-1/HO-1 axis activation. These findings indicate that Verapamil HCl could be a very effective treatment agent against PRRSV.

## INTRODUCTION

Porcine reproductive and respiratory syndrome virus (PRRSV) causes an acute infectious disease called PRRS, which is among the most economically damaging diseases affecting swine production worldwide. This syndrome manifests primarily as breeding complications in female pigs and as serious respiratory distress in both female and male pigs (1–3). As a member of the *Arteriviridae* family, PRRSV is an enveloped, positive-sense RNA virus. This pathogen exists in two primary forms: the European strain (PRRSV-1) and the North American strain (PRRSV-2), which share approximately 60% nucleotide similarity (4). Since the emergence of PRRSV in the late 1980s, numerous variants of this virus have been detected across various countries (5, 6). Given that PRRSV has a high mutation rate and strong immune evasion capabilities, the effectiveness of current vaccines and antiviral treatments against PRRSV remains limited (7–10). Therefore, novel anti-PRRSV agents must be developed urgently.

Verapamil HCl is an FDA-approved L-type calcium channel blocker primarily used to treat angina pectoris and supraventricular arrhythmias by antagonizing Ca^2+^ overload through the suppression of L-type channels (11). However, several studies have demonstrated that Verapamil HCl not only reduces inflammatory responses but also exerts antioxidant effects to mitigate tissue injury (12). Verapamil HCl has also been reported to inhibit infections caused by viruses such as the respiratory syncytial virus (13), Ebola virus (14), rhinovirus (15), and influenza A virus (16). However, the effectiveness of this drug and its definite mechanism of action against PRRSV remain unclear.

This study sought to assess the anti-PRRSV activities of Verapamil HCl and investigate the molecular pathways underlying its effects. Our findings revealed that Verapamil HCl successfully suppresses viral replication in both susceptible cell lines and in piglets. Further mechanistic analysis revealed that Verapamil HCl enhances antiviral interferon (IFN) responses and alleviates inflammation through the p38/nuclear factor erythroid-2-related factor 2 (Nrf2)/Kelch-like ECH-associated protein 1 (Keap1) signaling axis. Collectively, these results indicate that Verapamil HCl may serve as a viable therapeutic candidate for treating PRRSV infections.

## MATERIALS AND METHODS

### Viruses, cells, and chemicals

Laboratory stocks of two PRRSV-1 isolates, GZ11-G1 (GenBank: KF001144.1) and P073-3 (GenBank: MK214314.1), as well as five PRRSV-2 strains, including VR-2332 (GenBank: EF536003.1), CH-1a (GenBank: AY032626), SD16 (GenBank: JX087437.1), JXA1 (GenBank: EF112445.1), and NADC30-like (GenBank: KX766379), were used in this study. These strains were proliferated in MARC-145 cells, and their 50% tissue culture infectious dose (TCID_50_) was calculated using the Reed-Muench method. Briefly, MARC-145 cells were seeded in 96-well plates, and the viral supernatants from different samples were serially diluted 10-fold. Eight replicates were designed for each dilution (eight dilutions) and added to different wells with 100 μL. The cells were cultured at 37°C for 3–5 days, and the cytopathic effects were observed every day. The TCID_50_ value was calculated using GraphPad Prism version 8.0 (San Diego, CA, USA).

PRRSV-susceptible cell lines, namely MARC-145 and PK-15^CD163^ cells, were cultured in Dulbecco’s modified Eagle medium (DMEM) supplemented with 10% fetal bovine serum (FBS) and 1% of an antibiotic-antimycotic mixture (Servicebio, Wuhan, China). Meanwhile, porcine alveolar macrophages (PAMs) were isolated from 8-week-old PRRSV-negative piglets and maintained in RPMI-1640 containing 10% FBS and 1% of an antibiotic-antimycotic mixture. Briefly, the lungs from piglets were washed five times with phosphate-buffered saline (PBS), and the lavage was centrifuged at 1,000 × *g* and 4°C for 10 min. The cell pellet was resuspended in RPMI-1640 medium (Gibco, Invitrogen Corporation, CA, USA) containing 10% FBS, penicillin (100 units/mL), and streptomycin (100 µg/mL). All cells were incubated at 37°C under 5% CO_2_.

Verapamil HCl was purchased from Shandong Sparkjade Biotechnology Co., Ltd. (Jinan, China) and dissolved in dimethyl sulfoxide (DMSO) (MCE, NJ, USA). The concentration of the stock solution was 45 mM.

### Cell viability analysis

Cell counting kit-8 (CCK-8) was obtained from Beyotime Biotechnology (Nanjing, China) and used to detect the cytotoxicity of Verapamil HCl against MARC-145 cells, PK-15^CD163^ cells, and PAMs. Briefly, when these cells reached 90% confluence in 96-well plates, they were treated with different doses of Verapamil HCl (10, 20, 40, 60, 80, and 100 µM) for 24 h. Then, 100 µL DMEM or RPMI-1640 containing 10% CCK-8 reagent was added to each well, and the cells were incubated at 37°C for another 2 h. The viable cell count was assessed through absorbance measurements at 450 nm using a microplate reader (BioTek, USA). Cell viability was calculated as follows:

Cell survival rate (%) = [OD (sample) – OD (blank)/OD (control) – OD (blank)] × 100%.

### RNA extraction and quantitative real-time PCR (qPCR)

The qPCR assays were carried out as follows. Briefly, cell samples were collected at the indicated time points. Total RNA was extracted using the RNA Simple Total RNA Kit (TIANGEN, Beijing, China) and detected using an Epoch Micro-Volume spectrophotometer (BioTek, NJ, USA). Subsequently, total RNA (1 µg) was reverse transcribed into cDNA using the PrimeScript RT Master Mix (Takara, Beijing, China), and qPCR was performed using the 2× Universal SYBR Green qPCR Mix (Sparkjade, Jinan, China) with QuantStudio 1 Plus (ABI, CA, USA). Transcripts of the internal reference gene *GAPDH* were amplified to normalize the total RNA input. The relative levels of *ORF-7*, *Nrf2*, and *HO-1* were quantified using the 2^−∆∆C*t*^ method. All target gene primers used for qPCR are listed in Table 1.

**TABLE 1:** List of primers used in this study

Genes	Forward primer (5′–3′)	Reverse primer (5′–3′)
*ORF-7^a^*	ATGGCCGGTAAAAATCAGAGCC	TTAATTCGCACCCTGACTGG
*ORF-7^b^*	ATGCCAAATAACAACGGCAAGC	TCATGCTGAGGGTGATGCTGTG
HO-1	AGTTCATGAAGAACTTTCAGAA	TACCAGAAGGCCATGTCC
Nrf2	ATTCAATGATTCTGACTCTG	CGTATCCCCAGAAGAATGTA
siHO-1	GGTCCTCACACTCAGCTTT	AAAGCTGAGTGTGAGGACC
siNrf2	GACATTCCCATTTGTAGAT	ATCTACAAATGGGAATGTC
GAPDH	GTCTCCTCTGACTTCAACAGCG	ACCACCCTGTTGCTGTAGCCAA

^*a*^ Used for PRRSV-1.

^*b*^ Used for PRRSV-2.

In order to evaluate the copy number of PRRSV in the cell supernatant, the *ORF-7* gene fragment of PRRSV-1 and PRRSV-2 was inserted into a pMD18-T vector to generate the recombinant plasmids pMD18-T-ORF7-1 and pMD18-T-ORF7-2. Data obtained using these recombinant plasmids were used to generate a standard curve. Specifically, the standard curve was plotted based on the results of parallel PCRs performed using serial dilutions of standard DNA samples. The absolute quantities of supernatant RNA were calculated through normalization against the standard curve.

Such experiments were performed to assess virus inhibition, direct virucidal activity, phase of suppression during the PRRSV lifecycle, virus adsorption, virus internalization, virus replication, virus release, and PRRSV copy number in serum samples from piglets.

### Evaluation of virus inhibition

MARC-145 cells, PK-15^CD163^ cells, and PAMs were seeded into 12-well plates and cultured until they reached 80–90% confluence. Then, these cells were treated with Verapamil HCl (5, 10, 20, 40, and 80 µM) for 2 h, followed by 0.1 MOI SD16 for 1 h. The medium was then replaced with 3% FBS DMEM or 3% FBS RPMI-1640 containing the indicated concentrations of Verapamil HCl. The cells and cellular supernatants were harvested at 24 hpi for further qPCR and western blot assays.

### Western blot analysis

All cell samples were lysed using NP40 lysis buffer (Beyotime, Nanjing, China), and the protein concentrations were detected using the Pierce BCA Protein Assay Kit (Thermo, Waltham, USA). The protein lysates were mixed with 5× sodium dodecyl sulfate (SDS) sample loading buffer and separated using 12% sodium dodecyl sulfate-polyacrylamide gel electrophoresis. The protein bands were transferred to polyvinylidene difluoride (PVDF) membranes, which were blocked with 5% skimmed milk powder for 1 h. Subsequently, the membranes were incubated with the indicated primary antibodies, including anti-PRRSV-1 and PRRSV-2 N protein antibodies; anti-heme oxygenase 1 (HO-1) mouse mAb (Servicebio); anti-p38/p-p38 rabbit pAb (CST, MA, USA); anti-ERK1/2 and p-ERK1/2 rabbit pAbs (Servicebio); anti-JNK1 + JNK2 + JNK3 rabbit pAb (Servicebio); anti-p-JNK mouse antibody (Santa Cruz, CA, USA), anti-Nrf2 antibody (Servicebio), anti-Keap1 rabbit pAb (Servicebio), anti-Lamin B1 rabbit pAb (Servicebio), and anti-β-actin antibody (Abcam, Cambridge, UK). Subsequently, the membranes were treated with a secondary HRP-conjugated goat anti-mouse antibody or goat anti-rabbit antibody (Jackson, West Grove, PA, USA). Finally, specific bands were visualized using the ECL reagent (Thermo) on a ChemiDoc MP Imaging System (Bio-Rad, CA, USA). The protein molecular weight marker (EC1019) was purchased from Shandong Sparkjade Biotechnology Co., Ltd. (Jinan, China).

### Analysis of antiviral activity

To assess the direct virucidal activity of Verapamil HCl against PRRSV, MARC-145 cells were cultured in 12-well plates and grown to 90% confluence. PRRSV SD16 suspensions (MOI = 0.1, 0.5, and 1.0) were pre-incubated with 80 µM Verapamil HCl at 37°C for 2 h prior to inoculation in the 12-well plates for 1 h. Cell lysates and culture supernatants were harvested at 24 hpi for viral replication analysis via western blotting and qPCR assays.

To determine which phase of the PRRSV lifecycle Verapamil HCl targets, four distinct treatment protocols were established: T1 (continuous treatment), T2 (pre-infection treatment), T3 (concurrent treatment), and T4 (post-infection treatment). MARC-145 cells were cultured in 12-well plates and exposed to varying Verapamil HCl concentrations. The cells were subsequently challenged with PRRSV SD16 (MOI = 0.1), and viral replication was assessed through qPCR and western blot analysis.

### Virus adsorption assay

MARC-145 cells cultured in 12-well plates were pre-treated with either 80 µM Verapamil HCl or DMSO at 37°C for 1 h. The cells were then chilled on ice and exposed to PRRSV SD16 (MOI = 1) for 1 h, followed by two washes with ice-cold PBS to eliminate unattached virus particles. The quantity of cell-bound PRRSV was determined through qPCR analysis.

### Virus internalization assay

MARC-145 cells were cultured in 12-well plates and pre-conditioned with either 80 µM Verapamil HCl or DMSO control at 37°C for 1 h. The cells were then challenged with PRRSV SD16 (MOI = 1) at 4°C for 1 h to facilitate viral binding. Non-attached virus particles were eliminated through two washes with ice-cold PBS, and culture medium was replaced with DMEM supplemented with 3% FBS and either 80 µM Verapamil HCl or DMSO. The cells were subsequently incubated at 37°C for 1 h to allow viral internalization. Following incubation, the cells were treated with citrate buffer (pH 3.0) to remove surface-bound viruses, and internalized PRRSV particles were quantified using qPCR.

### Viral replication assay

MARC-145 cells were cultured in 12-well plates and challenged with PRRSV SD16 (MOI = 1) at 37°C. Extracellular virus particles were eliminated through two PBS washes at 6 hpi. Fresh DMEM supplemented with 3% FBS and either 80 µM Verapamil HCl or DMSO was added, and the cells were maintained at 37°C. At 7, 8, 10, and 16 hpi, cell samples were harvested for PRRSV quantification via qPCR.

### Virus release assay

MARC-145 cells were inoculated with PRRSV (MOI = 1) and incubated at 37°C for 1 h. Following initial infection, the culture medium was replaced with DMEM containing 3% FBS. At 24 hpi, the cells were subjected to three consecutive PBS washes before treatment with either 80 µM Verapamil HCl or DMSO vehicle control in DMEM containing 3% FBS for 30 or 60 min at 37°C. Culture supernatants were collected at designated time points and analyzed using qPCR to quantify the number of released virus particles.

### ELISA detection

Cell supernatants were harvested from PAM cultures treated with Verapamil HCl. After infection with PRRSV SD16 (MOI = 0.1) for 24 h, the IL-6, IL-8, IL1-β, TNF-α, IFN-α, and IFN-β levels in the supernatant were analyzed using porcine IL-6, IL-8, IL1-β, TNF-α, IFN-α, and IFN-β ELISA kits (JONLNBIO, Shanghai, China), respectively, based on the manufacturer’s instructions. In addition, the serum levels of IFN-α/β in different groups of piglets were detected at the indicated time points using similar commercial ELISA kits.

### Small interfering RNAs (siRNA) knockdown assay

The siRNAs designed to target Nrf2 and HO-1 were purchased from Gene Pharma Co., Ltd. (Shanghai, China). PAMs were transfected with siRNAs using the X-tremeGENE siRNA Transfection Reagent for a 12-h period. Following transfection, the cells were challenged with PRRSV at an MOI of 0.1 and cultured with or without 80 µM Verapamil HCl for 24 h. Non-targeting siRNA was employed as a negative control to account for non-specific effects. The expression levels of HO-1, Nrf2, and ORF-7 were subsequently assessed through qPCR and western blot assays using the methods described above.

### Ca^2+^ measurements

A Fluo-4 Calcium Flux Analysis Kit (MCE) was employed to detect cytoplasmic Ca^2+^ levels *in vitro* according to the manufacturer’s instructions. Briefly, MARC-145 cells were seeded into a 96-well cell plate overnight (Beyotime) at a density of 1 × 10^4^ cells/well. The cells were pre-incubated with Verapamil HCl (40 or 80 µM) for 2 h and then infected with PRRSV SD16 (MOI = 1) for 1 h. The medium was replaced with 3% FBS DMEM containing the indicated concentrations of Verapamil HCl. Meanwhile, KCl and BAPTA-AM were used for treatment in the positive and negative control groups, respectively. Finally, all cells were treated with 100 μL of Fluo-4 dye-loading solution at 24 hpi and incubated at 37°C for 0.5 h. Finally, the calcium flux was detected using a multi-functional microplate reader (Tecan Spark, Switzerland) at Ex/Em = 485/526 nm. Meanwhile, fluorescence signals were observed and imaged using the Leica DMi8 fluorescence microscope (Leica, Solms, Germany).

### Immunofluorescence assay (IFA)

MARC-145 cells were seeded onto coverslips in 12-well plates overnight, pre-incubated with Verapamil HCl (40 and 80 µM) for 2 h, and then infected with PRRSV SD16 (MOI = 1). KCl and BAPTA-AM were used for treatment in the positive and negative control groups, respectively. All cells were fixed with cold 75% ethanol at 24 hpi. Subsequently, the cells were incubated with porcine PRRSV convalescent serum (primary antibody) and probed with DyLight 594-conjugated AffiniPure Goat Anti-Swine IgG (H+L) (Jackson, PA, USA) secondary antibodies. Nuclear material was stained using 4′,6-diamidino-2-phenylindole (DAPI) (Beyotime). Finally, a Leica DMi8 fluorescence microscope (Leica) was employed for imaging.

### *In vivo* anti-PRRSV assay and pathological examination

Fifteen 6-week-old Landrace piglets purchased from Xilingjiao Breeding and Raising Center (free of PRRSV) were randomly divided into three groups (*n* = 5). Group 1 was exposed to PRRSV SD16 only, Group 2 was challenged with PRRSV SD16 in the presence of Verapamil HCl, and Group 3 was treated with DMSO. Group 1 and Group 2 piglets were challenged with the virus (3 × 10^5^ TCID_50_) via nasal drip. All piglets had free access to feed and water and were housed in separate rooms to prevent cross-contamination. Group 1 and Group 3 received MEM containing 0.1% DMSO (5 mL orally), and Group 2 received 20 mg/kg Verapamil HCl diluted in 5 mL of MEM (orally) on −1, 1, 3, 5 days post-challenge (DPC). Serum samples were collected on days 3, 7, 10, 14, and 21 after the virus challenge, and the copies of SD16 were detected using qPCR. Additionally, the levels of IFN-α and IFN-β were measured using ELISA. The clinical symptoms of all piglets were monitored daily. At 21 DPC, all piglets were euthanized via the intravenous injection of a lethal dose of sodium pentobarbital (Sinopharm). Immediately after euthanasia, necropsy was performed, and lung tissues were obtained from each piglet and fixed in 10% paraformaldehyde for histopathological examination.

To evaluate the protective effects of Verapamil HCl against PRRSV infection in piglet lungs, hematoxylin and eosin (H&E) staining was performed. Lung tissue samples from all experimental animals were fixed in 10% paraformaldehyde before dehydration using graded ethanol concentrations (70–100%). The specimens were subsequently cleared with xylene and processed for paraffin embedding before sectioning (4 µm thickness). The tissue sections were carefully placed on glass slides and processed via deparaffinization using xylene, followed by rehydration using decreasing ethanol concentrations. H&E staining was then applied for histopathological evaluation. Microscopic examination of the prepared slides was performed using a Leica DMi8 inverted microscope system.

### Statistical analysis

Data analysis was conducted using GraphPad Prism version 8.0 (San Diego, CA, USA). Between-group comparisons were conducted using unpaired Student’s *t*-tests. The results were considered statistically significant when *P* < 0.05, with significance levels indicated as follows: * *P* < 0.05, ** *P* < 0.01, and *** *P* < 0.001.

## RESULTS

### Verapamil HCl exerts inhibitory effects against PRRSV *in vitro*

**Fig 1:**
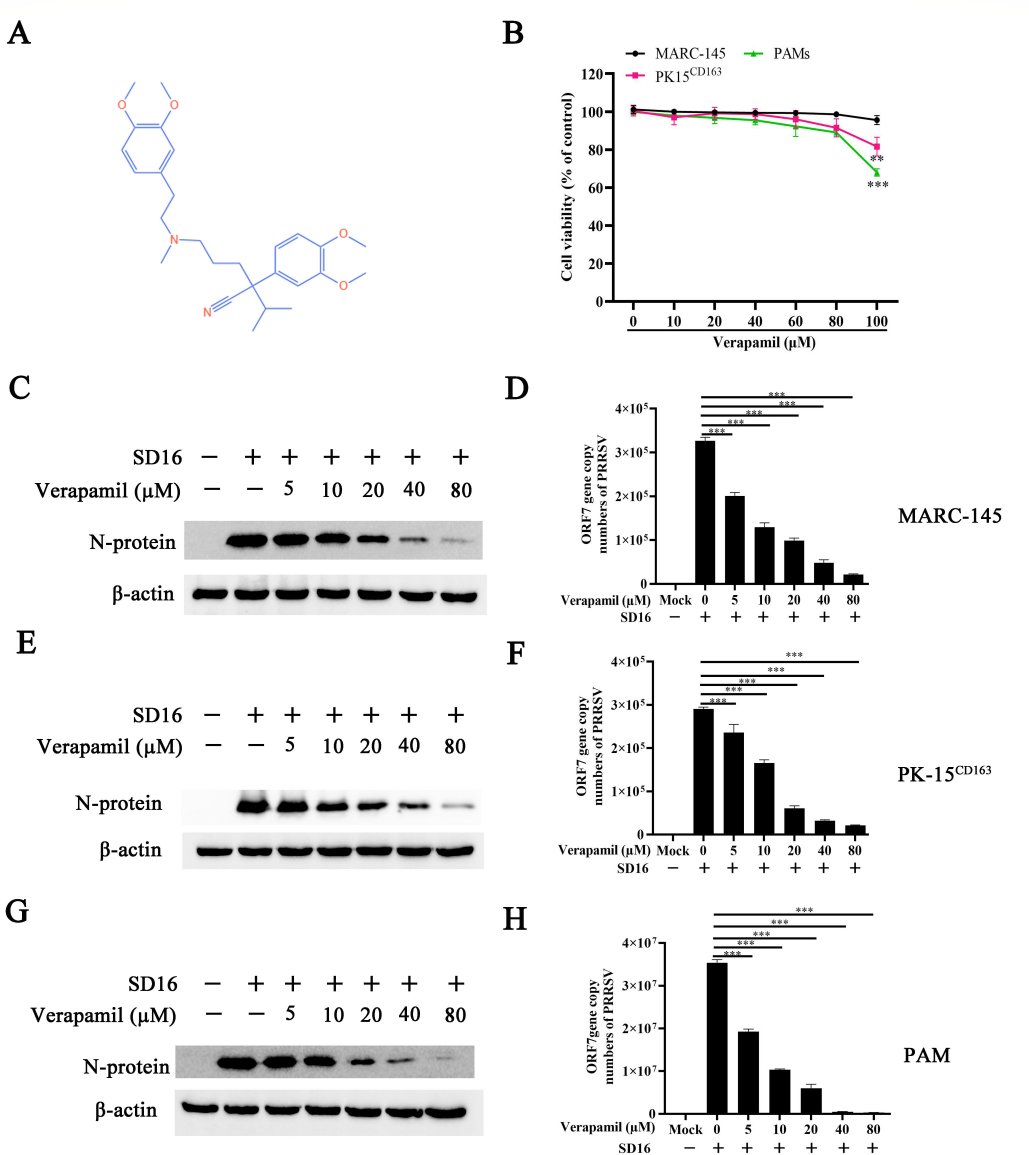
Concentration-dependent inhibition of PRRSV SD16 infection by Verapamil HCl in different susceptible cells. (**A**) Verapamil HCl chemical structure. (**B**) Assessment of Verapamil HCl cytotoxic effects on MARC-145 cells, PK-15^CD163^ cells, and PAMs. These cells were exposed to varying Verapamil HCl concentrations (10, 20, 40, 60, 80, and 100 μM) for 24 h, followed by viability determination using CCK-8 assay methodology. Cell viability calculations utilized untreated cells as reference controls (designated as 100% viability). MARC-145 cells (**C and D**), PK-15^CD163^ cells (**E and F**), and PAMs (**G and H**) underwent PRRSV SD16 challenge (MOI = 0.1) following pre-treatment with Verapamil HCl (5, 10, 20, 40, and 80 µM). Culture medium was subsequently replaced with 3% FBS DMEM or RPMI-1640 containing Verapamil HCl at indicated concentrations. Cellular samples and culture supernatants were harvested at 24 hpi for N protein expression analysis via western blot methodology and progeny virus quantification using qPCR methodology. β-actin was employed as the internal loading reference. Results represent data from three independent experimental replicates analyzed using Student’s *t*-test statistical analysis. ***P < 0.001: statistical significance relative to 0 µM Verapamil HCl control treatments.

The molecular structure and physicochemical characteristics of Verapamil HCl are depicted in Fig. 1A. Cytotoxicity evaluations were conducted using CCK-8 assays to determine the safe concentration range of Verapamil HCl in different PRRSV-susceptible cells, namely MARC-145 cells, PK-15^CD163^ cells, and PAMs. Figure 1B demonstrates that the highest tested concentration of Verapamil HCl (80 µM) produced no detectable effects on cell viability across all tested cell lines.

The potential antiviral effects of Verapamil HCl were evaluated through systematic experimental approaches. MARC-145 cells, PK-15^CD163^ cells, and PAMs were pretreated with different concentrations of Verapamil HCl before a PRRSV SD16 challenge (MOI = 0.1). Subsequently, both cellular and supernatant fractions were obtained for viral replication assessment via western blot and qPCR. The experimental data indicated that Verapamil HCl suppresses PRRSV N protein expression and reduces progeny virus production in MARC-145 cells, demonstrating dose-dependent inhibitory effects (Fig. 1C and D). Comparable antiviral efficacy was also observed in PK-15^CD163^ cells (Fig. 1E and F) and PAMs (Fig. 1G and H).

### Broad-spectrum activity against PRRSV-1 and PRRSV-2 variants

**Fig 2:**
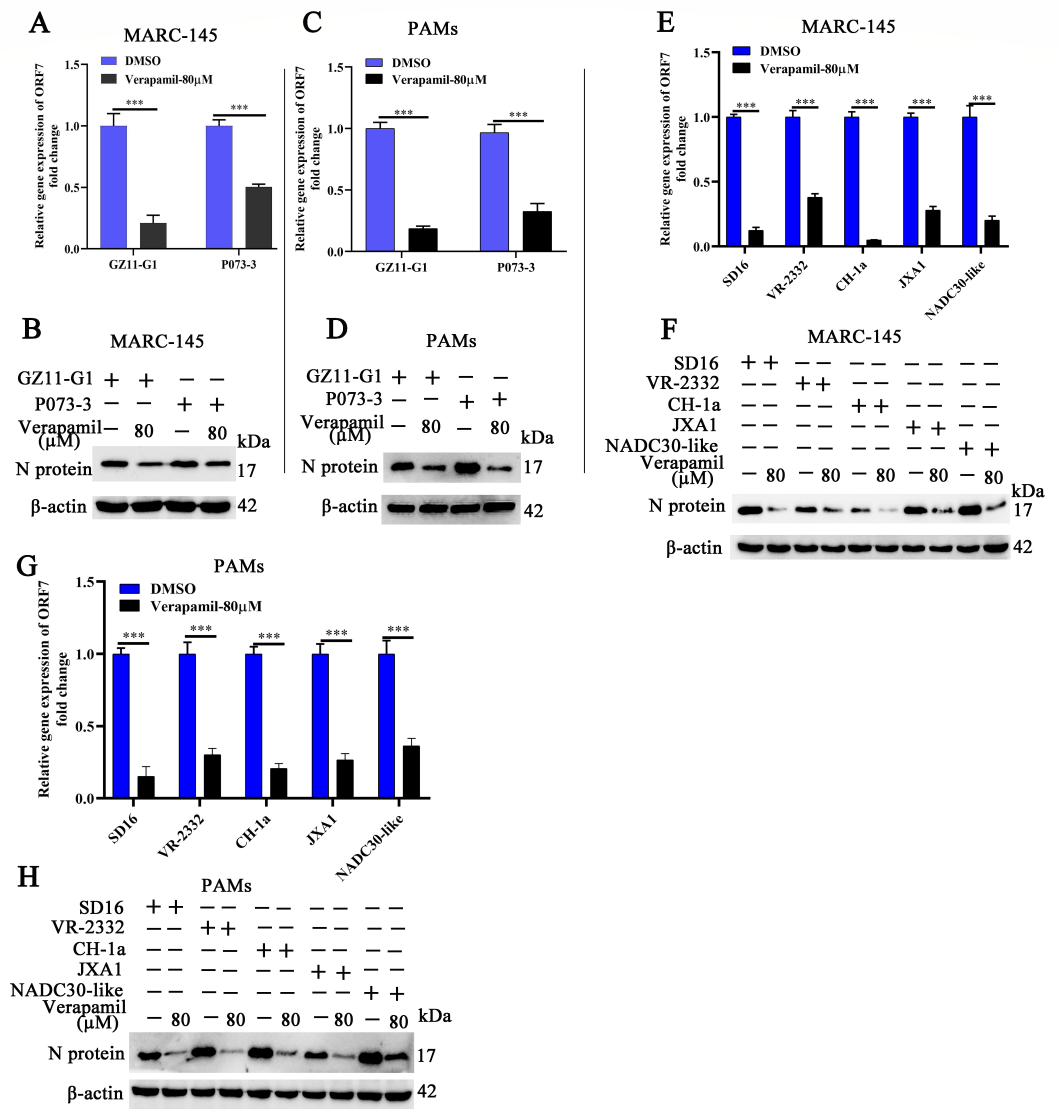
Verapamil HCl broad-spectrum anti-PRRSV activity. MARC-145 cells (**A and B**) and PAMs (**C and D**) underwent pre-treatment with 0 or 80 µM Verapamil HCl for 2 h. Subsequently, the cells were challenged with distinct PRRSV-1 strains (GZ11-G1 or P073-3) at 0.1 MOI for 1 h at 37°C. Following 24 hpi, cellular samples were processed to evaluate PRRSV N protein expression via qPCR and western blot methodologies, respectively. (**E and F**) MARC-145 cells received preconditioning with 0 or 80 µM Verapamil HCl for 2 h, then further exposed to various PRRSV-2 strains (SD16, VR-2332, CH-1a, JXA1, and NADC30-like) at 0.1 MOI for 1 h. Cellular harvesting occurred at 24 hpi for quantification of PRRSV N mRNA and protein levels using qPCR and western blot analysis, respectively. (**G and H**) Additionally, Verapamil HCl antiviral efficacy against multiple PRRSV-2 strains was evaluated in PAMs. These cells underwent analysis for PRRSV N expression at 24 hpi via qPCR (**G**) and western blot (**H**) approaches. GAPDH functioned as the normalization reference gene, whereas β-actin provided loading control standards. Results demonstrate findings from three independent experimental replicates. ***P < 0.001: statistical significance versus DMSO control treatments (0 µM Verapamil HCl).

The antiviral spectrum of Verapamil HCl was examined against various PRRSV-1 strains (GZ11-G1 and P073-3) as well as PRRSV-2 strains (SD16, VR-2332, JXA1, CH-1a, and NADC30-like) through *in vitro* assays. MARC-145 cells and PAMs were exposed to Verapamil HCl (80 µM) in conjunction with the GZ11-G1 and P073-3 strains, respectively. Analysis at 24 hpi revealed a significant reduction of PRRSV-1 *ORF-7* mRNA levels in Verapamil HCl-treated MARC-145 cells and PAMs when compared to corresponding DMSO-treated controls (Fig. 2A and C). A concordant decrease in GZ11-G1 and P073-3 N protein expression was also detected following Verapamil HCl treatment (Fig. 2B and D).

Additional experiments were conducted by treating MARC-145 cells and PAMs with Verapamil HCl (80 µM) alongside multiple PRRSV-2 strains. The qPCR and western blot analyses demonstrated a substantial reduction in ORF-7 expression across all tested PRRSV-2 strains (SD16, VR-2332, JXA1, CH-1a, and NADC30-like strains) in MARC-145 cells following Verapamil HCl treatment (Fig. 2E and F). PAMs exhibited similar inhibitory responses to different PRRSV-2 strains after treatment with Verapamil HCl (Fig. 2G and H). These findings demonstrated that Verapamil HCl exhibits broad-spectrum antiviral activity against a diverse range of PRRSV strains.

### Verapamil HCl shows antiviral activity against PRRSV at multiple stages

**Fig 3:**
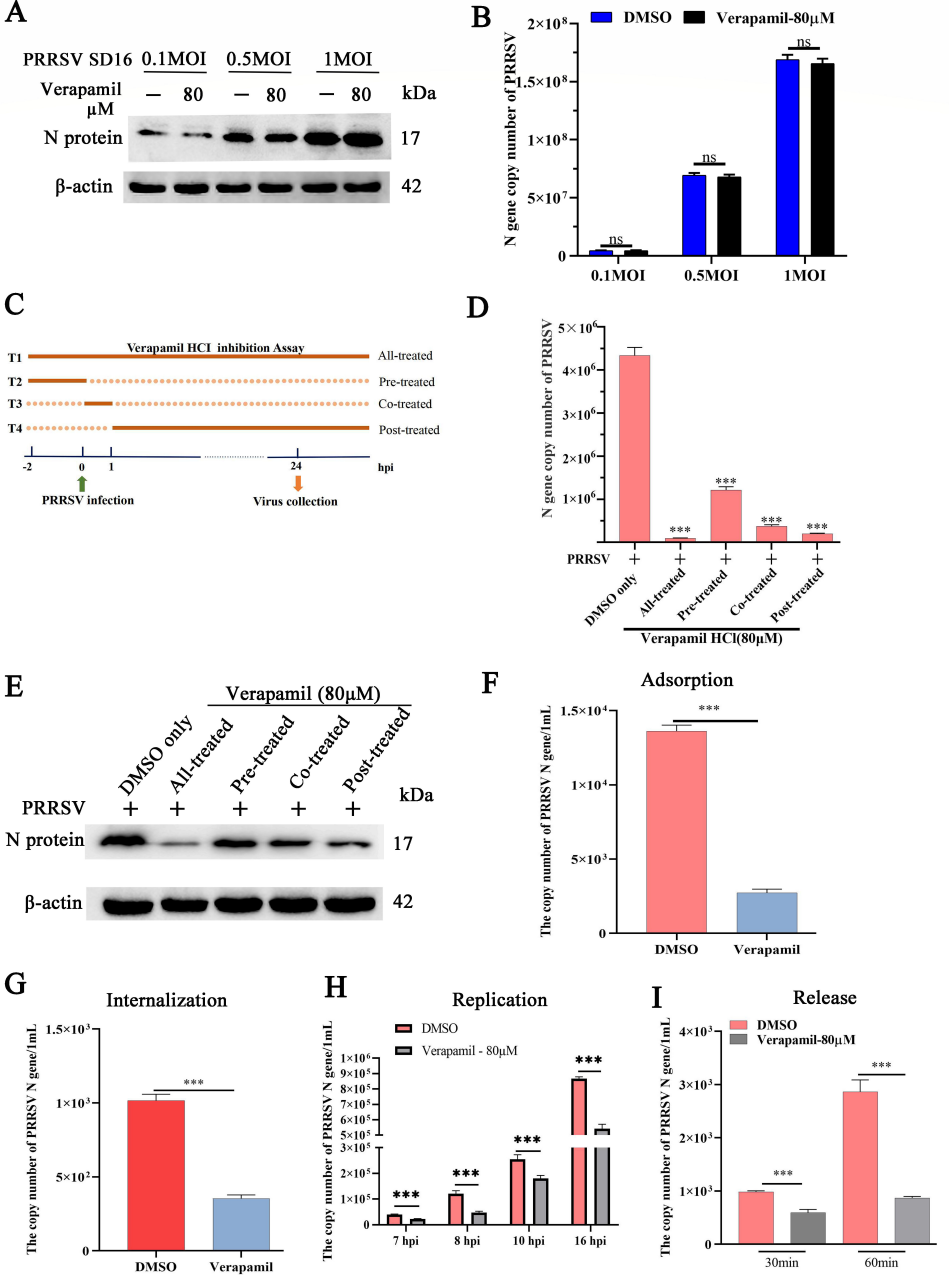
Mapping the stage of Verapamil HCl action during the PRRSV life cycle. PRRSV SD16 (MOI = 0.1, 0.5, or 1) was pre-incubated with Verapamil HCl (80 µM) or DMSO for 2 h at 37°C. Then, this mixture was inoculated into MARC-145 cells. Western blot (**A**) and qPCR (**B**) were employed to evaluate the virucidal activity of Verapamil HCl at 24 hpi. (**C**) Time-of-addition schematic, showing that MARC-145 cells were treated with Verapamil HCl (80 µM) at different time points relative to PRRSV SD16 (MOI＝0.1) infection, including pre-treatment, co-treatment, post-treatment, and all stage-treatment. The cellular supernatants and cells were collected at 24 hpi to analyze progeny virus generation using qPCR (**D**) and detect N protein expression using western blot (**E**). (**F**) In the adsorption assay, MARC-145 cells were pretreated with Verapamil HCl for 1 h at 37°C and washed with PBS. After incubation with 1 MOI PRRSV SD16 on ice for 1 h, the copy number of virus particles was tested using qPCR. (**G**) In the internalization assay, MARC-145 cells were pretreated with 80 µM Verapamil HCl for 1 h at 37°C and then incubated with PRRSV (MOI = 1) at 4°C for 1 h. The cells were washed and finally incubated with Verapamil HCl (80 µM) or DMSO for another 1 h at 37°C. The copy number of internalized virus particles was detected using qPCR at 24 hpi. (**H**) In the replication assay, MARC-145 cells were infected with PRRSV SD16 (MOI = 1) for 6 h, and the medium was then replaced with 3% FBS DMEM containing Verapamil HCl (80 µM) or DMSO. At 7, 8, 10, and 16 hpi, the mRNA levels of the PRRSV N protein were measured using qPCR. (**I**) For the release assay, MARC-145 cells were infected with PRRSV SD16 (MOI = 1) for 24 h, washed with PBS, and then incubated with 3% FBS DMEM containing 80 µM Verapamil HCl or DMSO. Supernatants were harvested to detect the number of released virus particles at 30 and 60 min using qPCR. The data shown are representative of three independent experiments. ***P < 0.001: compared with DMSO-treated cells and ns: not significant.

The direct virucidal activity of Verapamil HCl was investigated by exposing MARC-145 cells to premixed combinations of 80 µM Verapamil HCl and PRRSV SD16 at MOI= 0.1, 0.5, and 1. The subsequent analysis of cellular and supernatant samples revealed no significant differences in PRRSV N protein expression between Verapamil HCl-treated and DMSO-treated groups (Fig. 3A). Similarly, progeny virus counts remained comparable between the treatment groups (Fig. 3B), indicating that Verapamil HCl lacks direct virucidal action against PRRSV.

Time-of-addition experiments were conducted to identify the specific viral life cycle stages affected by Verapamil HCl. Cell populations were stratified into groups T1–T4 based on treatment timing—all-stage treatment, pre-treatment, co-treatment, and post-treatment—as illustrated in Fig. 3C. All cells were harvested at 24 hpi to evaluate PRRSV replication. The results demonstrated significantly lower viral copy numbers in all treatment groups (T1–T4) than in the DMSO control group, illustrating the multi-stage inhibitory activity of Verapamil HCl (Fig. 3D). In line with these findings, the suppression of N protein expression was also observed across MARC-145 cell populations (Fig. 3E).

Overall, stage-specific analysis revealed that Verapamil HCl effectively reduces PRRSV copy numbers during the adsorption (Fig. 3F), internalization (Fig. 3G), replication (Fig. 3H), and release phases (Fig. 3I). These findings demonstrated that Verapamil HCl perturbs PRRSV infection across multiple stages of the viral life cycle.

### Verapamil HCl suppresses early-stage viral infections by decreasing Ca^2+^ uptake

**Fig 4:**
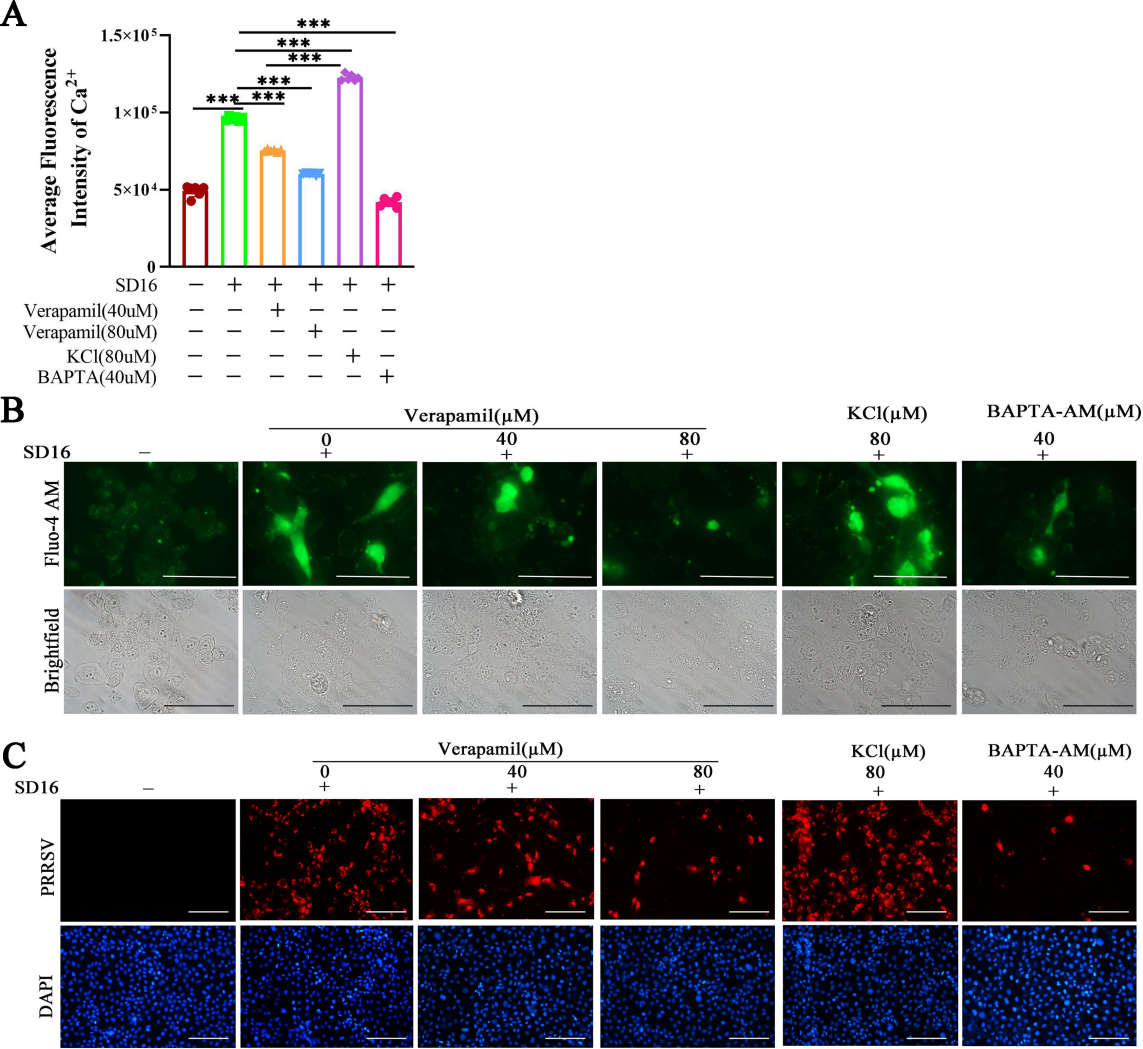
Verapamil HCl inhibits PRRSV infection by blocking Ca^2+^ influx. MARC-145 cells were seeded into 96-well cell plates and pre-incubated with Verapamil HCl (40 µM or 80 µM) for 2 h. The cells were then infected with 1 MOI PRRSV SD16 for 1 h, with KCl (80 µM) and BAPTA (40 µM) serving as controls. Finally, all cells were treated with Fluo-4 dye-loading solution (100 μL) at 24 hpi and incubated at 37°C for 0.5 h. Calcium flux was detected using a multi-functional microplate reader (**A**), and images were captured using a Leica DMi 8 fluorescence microscope (**B**). Here, green fluorescence indicated the presence of calcium (scale bar: 100 μm). (**C**) Meanwhile, an indirect immunofluorescence assay was performed to detect PRRSV protein expression (red). The nucleocapsid was stained with DAPI (blue) (scale bar: 100 µm). The data shown are representative of three independent experiments. ***P < 0.001: compared with PRRSV-infected cells.

Calcium ions (Ca^2+^) function as critical secondary messengers in mammalian cells, orchestrating diverse processes such as cell survival, pathogen recognition, metabolic regulation, signal transduction, apoptosis, and immune activation (17–19). Previous investigations have demonstrated that in susceptible cells, PRRSV exploits Ca^2+^ influx through L-type calcium channels to enhance viral replication (20, 21). Given that Verapamil HCl is an established L-type calcium channel antagonist with documented antiarrhythmic, antianginal, and antihypertensive properties (22), we investigated whether its anti-PRRSV mechanism involves Ca^2+^ homeostasis modulation. To this end, MARC-145 cells were treated with various Verapamil HCl concentrations prior to PRRSV infection. Intracellular Ca^2+^ levels were quantified using absorbance measurements and fluorescence microscopy. Verapamil HCl treatment was found to significantly reduce calcium fluorescence intensity (Fig. 4A), and concordant observations were also obtained through fluorescent calcium probe imaging (Fig. 4B). Additionally, substantial reductions in PRRSV protein expression were documented (Fig. 4C). These results confirmed that Verapamil HCl suppresses early-stage PRRSV infection through Ca^2+^ uptake inhibition.

### Verapamil HCl mitigates PRRSV-induced inflammation

**Fig 5:**
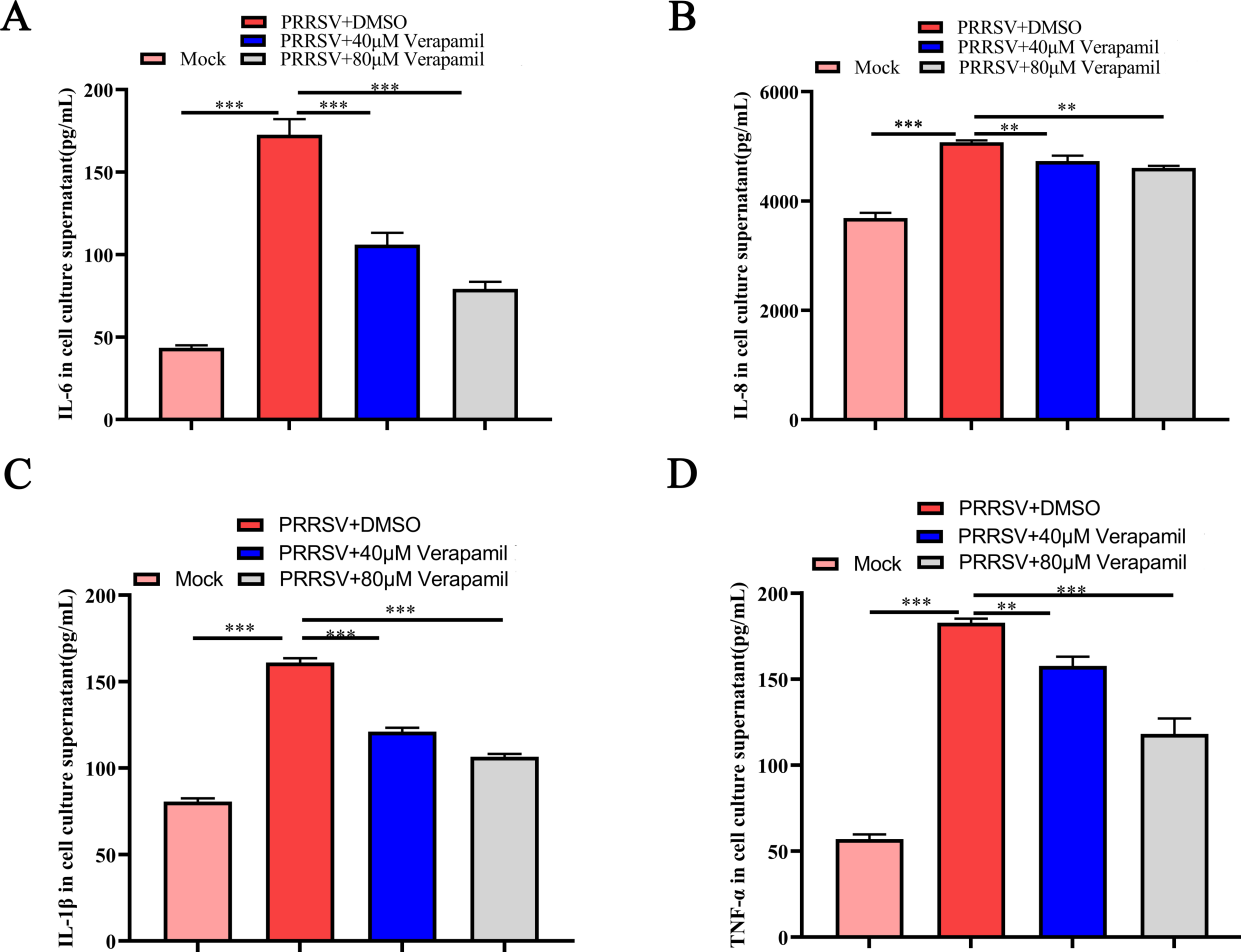
Verapamil HCl antagonizes PRRSV-induced inflammatory responses in PAMs. PAMs were treated with 40 or 80 µM Verapamil HCl in the presence of PRRSV SD16 (MOI = 0.1). The production of IL-6 (**A**), IL-8 (**B**), IL-1β (**C**), and TNF-α (**D**) at 24 hpi was detected using ELISA. The data shown are representative of three independent experiments. **P < 0.01 and ***P < 0.001: compared with DMSO + PRRSV-treated cells.

PRRSV infection stimulates the expression of pro-inflammatory cytokines, including IL-6, IL-1β, IL-8, and TNF-α, in susceptible cells. This facilitates viral replication *in vitro* and contributes to disease pathogenesis in swine (23–26). To evaluate the effects of Verapamil HCl on PRRSV-induced inflammatory cascades, PAMs were challenged with 0.1 MOI PRRSV SD16 in the presence of 40 or 80 µM Verapamil HCl. ELISA-based supernatant analysis at 24 hpi revealed that Verapamil HCl can significantly attenuate the PRRSV-induced production of pro-inflammatory mediators such as IL-6, IL-8, IL-1β, and TNF-α in a concentration-dependent manner (when compared to DMSO-treated PRRSV-infected controls) (Fig. 5). These observations indicated that Verapamil HCl ameliorates the pro-inflammatory responses triggered by PRRSV infection.

### Verapamil HCl suppresses PRRSV replication via Nrf2/HO-1 activation

**Fig 6:**
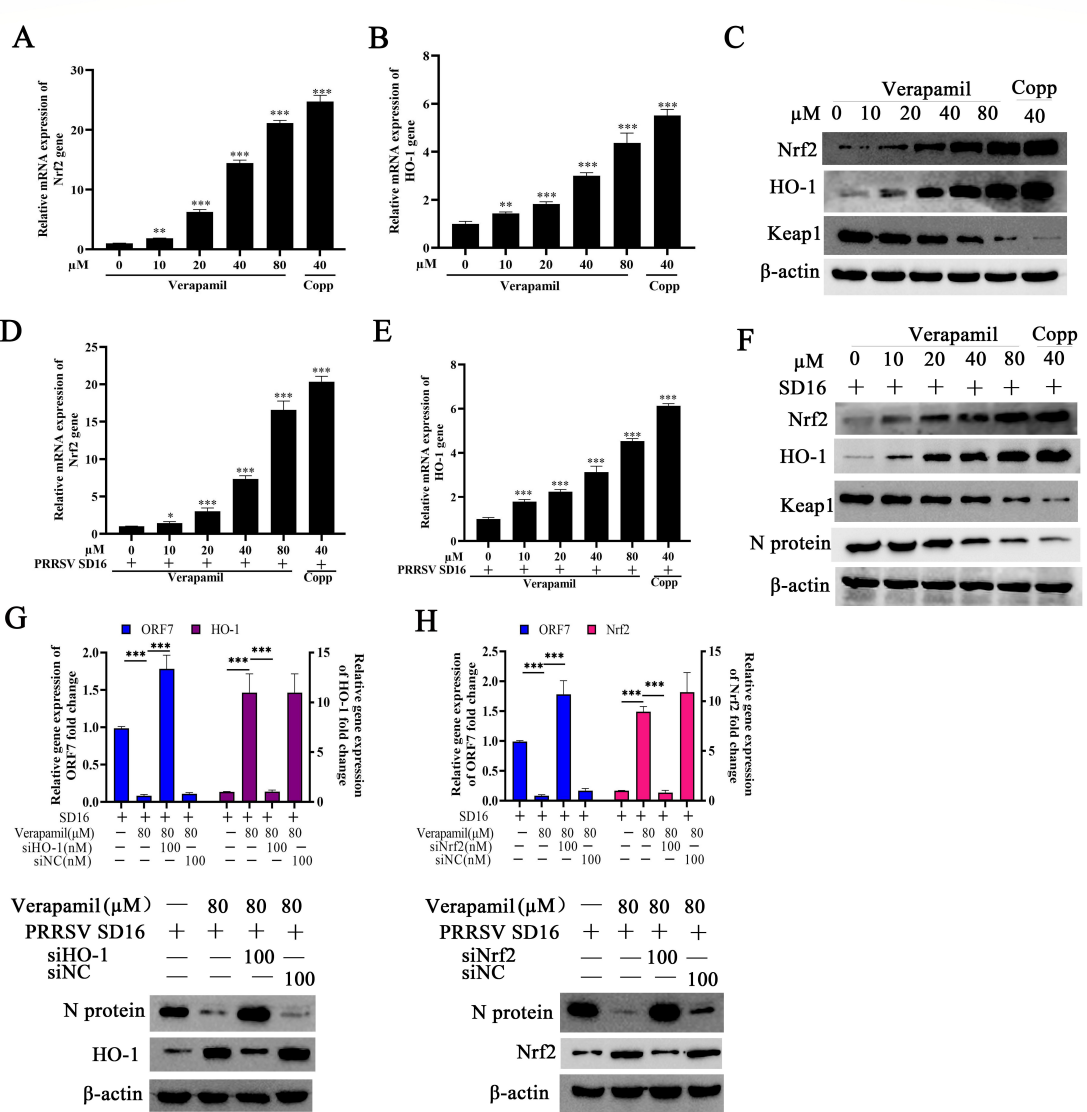
Verapamil HCl upregulates Nrf2/HO-1 expression to suppress PRRSV replication. (**A–C**) PAMs were treated with various concentrations of Verapamil HCl (10, 20, 40, and 80 µM) or cobalt protoporphyrin (CoPP) (40 µM) for 24 h and then harvested to extract RNA and protein. Nrf2, HO-1, and Keap1 expression was further analyzed using qPCR and western blot. (**D–F**) PAMs were treated with various concentrations of Verapamil HCl (10, 20, 40, and 80 µM) or CoPP (40 µM) for 2 h. Subsequently, these cells were subjected to PRRSV SD16 infection (MOI = 0.1) for 1 h and then harvested to detect HO-1, Nrf2, and Keap1 expression at the mRNA and protein levels using qPCR and western blot. (**G and H**) PAMs were transfected with siHO-1/siNrf2 or siNC and then infected with PRRSV SD16 in the presence or absence of Verapamil HCl (80 µM) for 24 h. HO-1, Nrf2, and ORF-7 expression levels were tested using qPCR and western blot. GAPDH served as the internal control, and β-actin served as the loading control. The data shown are representative of three independent experiments. *P < 0.05, **P < 0.01, and ***P < 0.001: compared with 0 µM Verapamil HCl-treated cells.

Verapamil HCl has previously been documented to exhibit antiviral, anti-inflammatory, and antioxidant activities, demonstrating therapeutic utility for angina, unstable angina, and hypertension management (13, 27). The transcription factor Nrf2 serves as a pivotal protective mechanism against exogenous and endogenous stressors in cells, including inflammatory responses and oxidative damage (28, 29). To investigate whether Verapamil HCl modulates Nrf2 expression, PAMs were treated with varying Verapamil HCl concentrations for 24 h, with or without a PRRSV SD16 challenge. The analysis of Nrf2, HO-1, and Keap1 expression revealed a significant upregulation of both the mRNA and protein levels of Nrf2 and HO-1 following Verapamil HCl treatment in PAMs. Meanwhile, Keap1 protein levels demonstrated marked downregulation (Fig. 6A through C). Similar patterns were observed in PAMs treated with both Verapamil HCl and PRRSV SD16, characterized by increased Nrf2 and HO-1 expression alongside decreased Keap1 levels (Fig. 6D through F). Finally, siRNA knockdown experiments were performed to validate the role of the Nrf2/HO-1 pathway in the effects of Verapamil HCl. The results demonstrated that siNrf2 and siHO-1 could effectively reverse the anti-PRRSV effects of Verapamil HCl in PAMs (Fig. 6G and H), confirming the mechanistic importance of this signaling pathway.

### Verapamil HCl shows anti-PRRSV activity via HO-1-mediated IFN production

Accumulating evidence has established HO-1 as a fundamental regulator of innate immune responses with antiviral effects, which are partially mediated through type I interferon (IFN-α/β) production (30, 31). Type I IFN responses represent critical components of antiviral innate immunity in virus-infected host cells (32). Therefore, we investigated whether Verapamil HCl induces type I IFN-mediated antiviral responses in PRRSV-susceptible cells.

**Fig 7:**
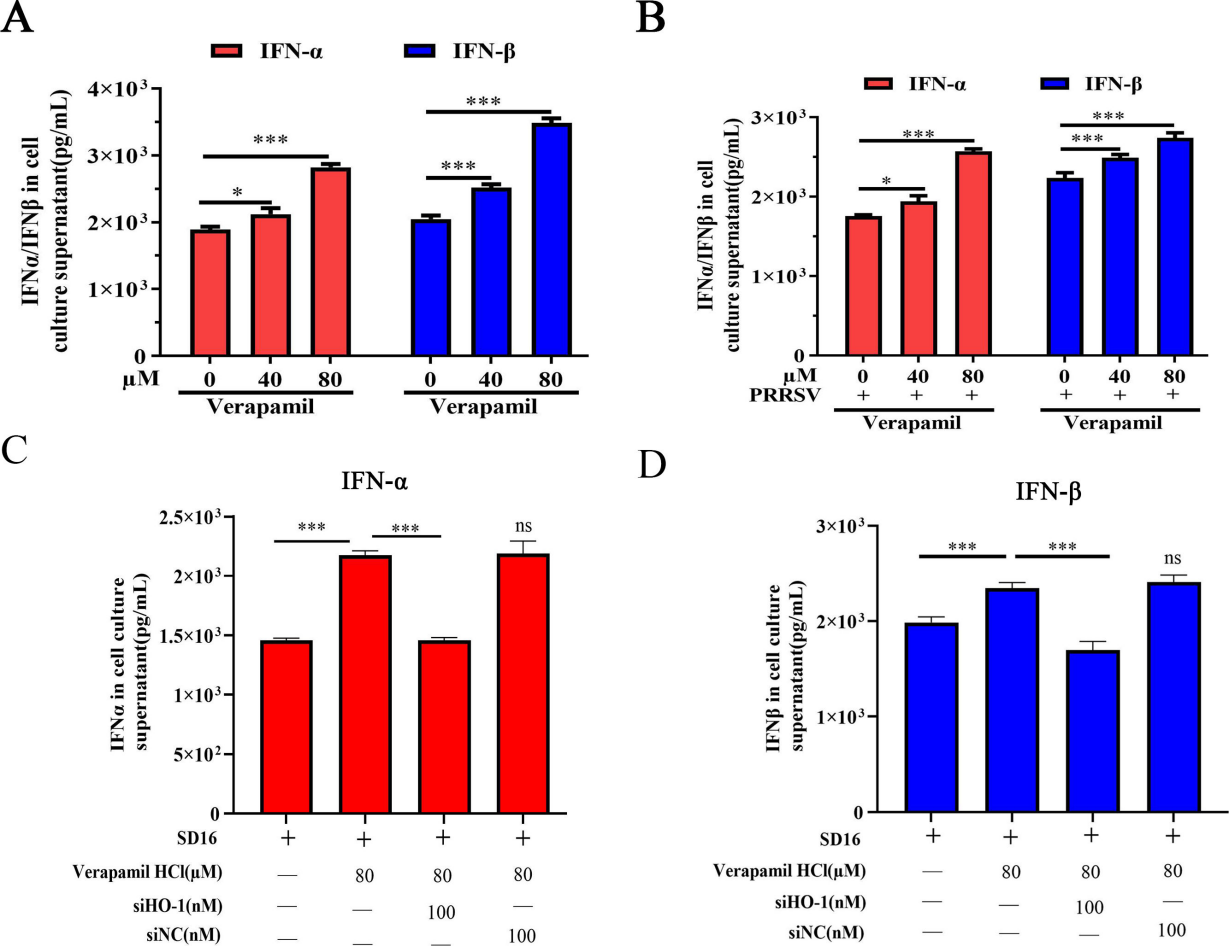
Verapamil HCl exerts anti-PRRSV effects by inducing an IFN-α/β antiviral response through HO-1 expression. PAMs were pretreated with Verapamil HCl (40 or 80 µM) for 2 h and then incubated without (**A**) or with (**B**) PRRSV SD16 (MOI = 0.1 MOI). The levels of IFN-α/β in the supernatant were detected using ELISA at 24 hpi. PAMs were transfected with siHO-1 for a 12-h period. Following transfection, the cells were challenged with PRRSV at an MOI of 0.1 and cultured with or without 80 µM Verapamil HCl treatment for 24 h. Non-targeting siRNA was employed as a negative control to account for non-specific effects. Cell supernatants were harvested to detect the IFN-α (**C**) and IFN-β (**D**) production in the supernatant. The data shown are representative of three independent experiments. *P < 0.05, ***P < 0.001, and ns: not significant: compared with 0 µM Verapamil HCl-treated cells or 80 µM Verapamil HCl-treated cells.

ELISAs were conducted to examine IFN-α/β production in PAMs under various PRRSV infection and Verapamil HCl treatment conditions. The data demonstrated that Verapamil HCl enhanced IFN-α/β generation in a concentration-dependent manner, both in uninfected (Fig. 7A) and PRRSV-infected cells (Fig. 7B). Given that PRRSV is recognized as a poor inducer of IFN responses in host cells and swine (33, 34), the data suggested that Verapamil HCl can counteract PRRSV-mediated type I IFN suppression. Interestingly, siHO-1 attenuated the IFN-α/β upregulation induced by Verapamil HCl (Fig. 7C and D). These results showed that Verapamil HCl induces type I IFN-mediated antiviral responses via HO-1 in PRRSV-susceptible cells.

### Verapamil HCl inhibits PRRSV proliferation via p38/Nrf2/HO-1 signaling activation

**Fig 8:**
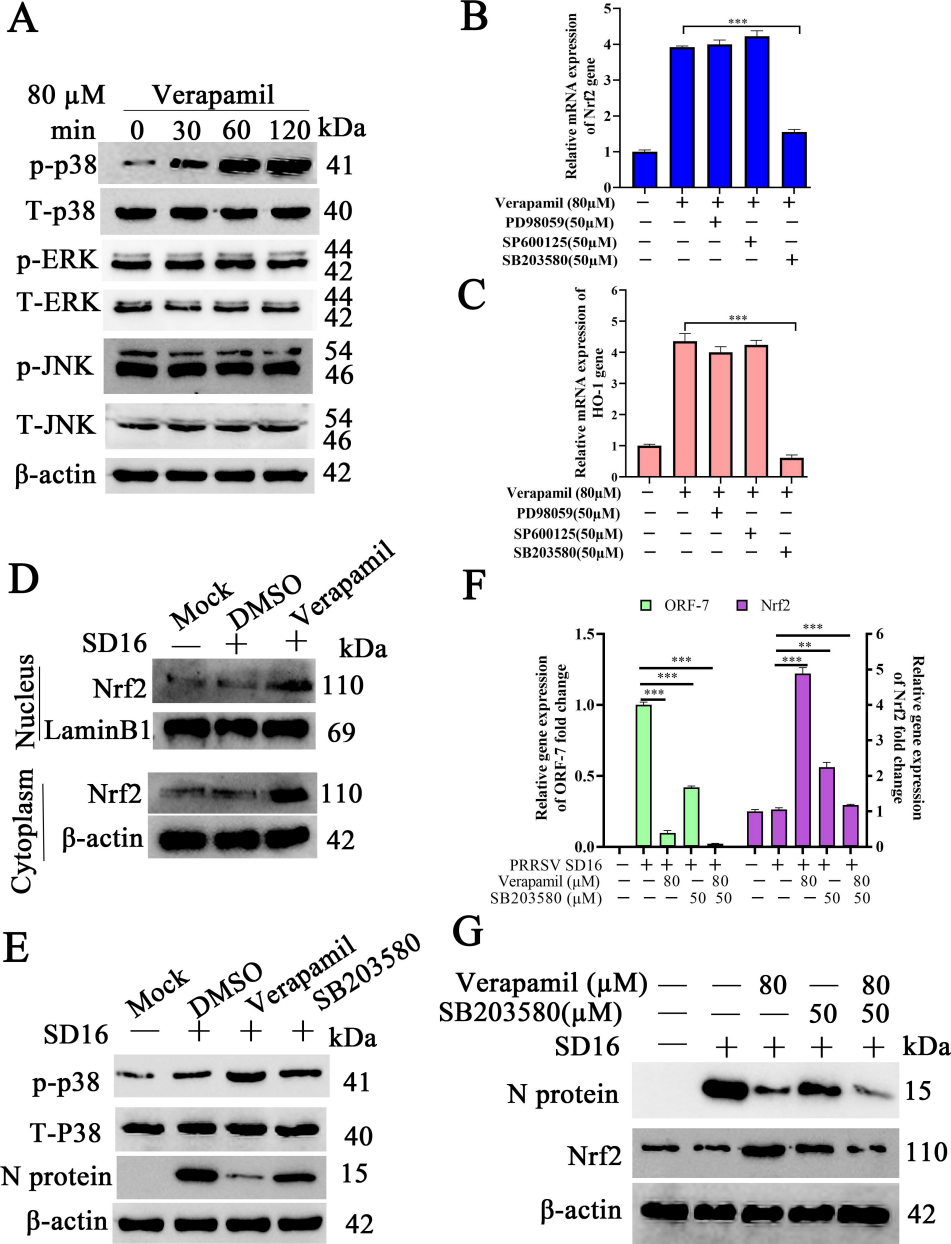
Verapamil HCl activates the p38/Nrf2/HO-1 cell signaling pathway. (**A**) MARC-145 cells were incubated with Verapamil HCl (80 µM) and collected at different time points (0, 30, 60, and 120 min) to detect the phosphorylated and total protein levels of p38, ERK1/2, and JNK using western blot. MARC-145 cells were incubated with a mixture of Verapamil HCl and a JNK inhibitor (SP600125), ERK1/2 inhibitor (PD98059), or p38 inhibitor (SB203580) for 2 h, and these cells were harvested to analyze *Nrf2* (**B**) and *HO-1* (**C**) expression using qPCR. (**D**) MARC-145 cells were pretreated with 80 µM Verapamil HCl for 2 h and then infected with 0.1 MOI PRRSV SD16 for 1 h. The protein expression levels of Nrf2 in the nuclear and cytoplasmic fractions were analyzed using western blot at 24 hpi. Lamin B1 and β-actin served as the loading controls. (**E**) Meanwhile, the amount of p-38, p-p38, and N protein expression in the total cellular fraction was also determined using western blot. MARC-145 cells were incubated with a mixture of Verapamil HCl (80 µM) and a p38 inhibitor (SB203580) for 2 h prior to infection with PRRSV SD16 (MOI = 0.1). The Nrf2 and N protein expression in these cells was analyzed at 24 hpi using qPCR (**F**) and western blot (**G**). GAPDH served as the internal control, and β-actin acted as the loading control. All the data shown are representative of three independent experiments. **P < 0.01 and ***P < 0.001.

The mitogen-activated protein kinases (MAPKs) p38, JNK, and ERK1/2 are signaling pathway components that regulate cellular defense mechanisms against pathogen invasion (35, 36). To determine whether MAPK pathways mediate the anti-PRRSV activity of Verapamil HCl, MARC-145 cells were pretreated with 80 µM Verapamil HCl for 2 h, followed by western blot analysis for p38, ERK1/2, and JNK expression. The results showed that p38 phosphorylation increased in a time-dependent manner following Verapamil HCl treatment (Fig. 8A). Conversely, ERK1/2 and JNK phosphorylation exhibited no significant changes at any analyzed time point. To further confirm the role of p38 in Verapamil HCl-mediated Nrf2 induction, MARC-145 cells were incubated with various MAPK inhibitors alongside Verapamil HCl. The p38 inhibitor SB203580 significantly suppressed Verapamil HCl-induced Nrf2 (Fig. 8B) and HO-1 (Fig. 8C) expression. However, PD98059 (ERK1/2 inhibitor) and SP600125 (JNK inhibitor) caused no significant alterations in Nrf2 and HO-1 expression levels.

Western blot analysis confirmed that Verapamil HCl enhanced overall Nrf2 expression and promoted the nuclear translocation of Nrf2 (Fig. 8D). Enhanced p38 phosphorylation was also observed in Verapamil HCl-treated cells (Fig. 8E). MARC-145 cells were treated with SB203580 and Verapamil HCl, followed by PRRSV SD16 infection. Subsequently, elevated PRRSV N mRNA and protein expression was observed under this condition (compared to Verapamil HCl monotherapy) (Fig. 8F and G), indicating that SB203580 could reverse the anti-PRRSV effects of Verapamil HCl. These findings confirmed that the antiviral activity of Verapamil HCl is dependent on p38-mediated Nrf2 pathway activation.

### Verapamil HCl decreases PRRSV replication and attenuates PRRSV-induced pathological damage in piglet lungs

**Fig 9:**
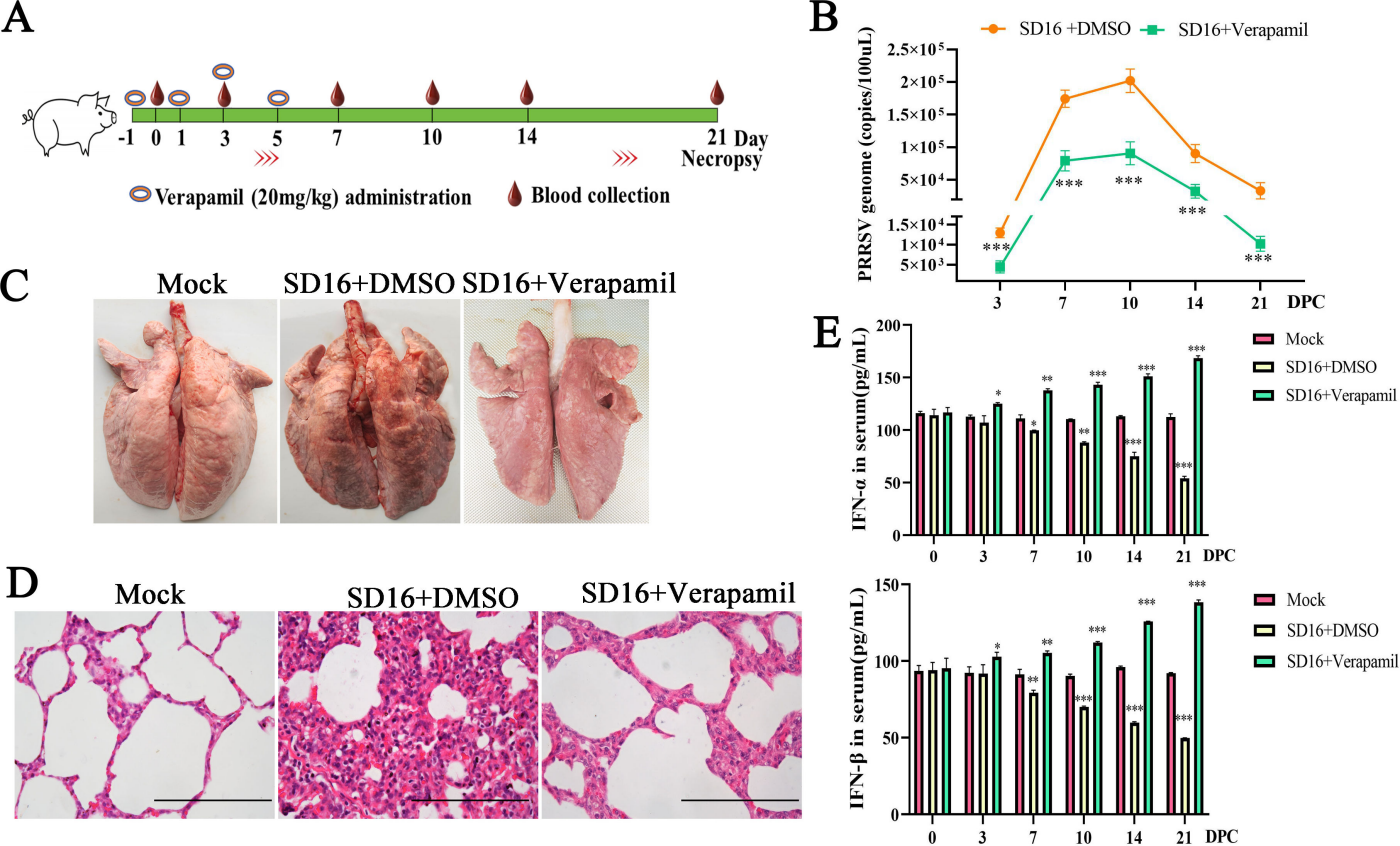
Verapamil HCl protects against PRRSV replication in piglets. The schematic showing the protocol of the PRRSV challenge experiment in piglets (**A**). PRRSV-negative piglets were randomly divided into three groups (*n* = 5/group): the Mock group, the PRRSV SD16+DMSO infected group, and the PRRSV SD16 + Verapamil HCl (20 mg/kg) treated group. Virus copy numbers detected in pig serum at different time points following PRRSV infection using qPCR (**B**). The results are presented as the mean ± standard deviation (error bars). ***P < 0.001: compared with SD16+DMSO treated piglets. All piglets were euthanized at 21 DPC. The pathological changes in the lungs were observed (**C**), and histopathological analysis was performed with HE staining (scale bar: 100 µm) (**D**). The serum levels of IFN-α/β in each group were detected at indicated time points using commercial kits (**E**). *P < 0.05, **P < 0.01, and ***P < 0.001: compared with the Mock group.

To assess the anti-PRRSV activity of Verapamil HCl *in vivo*, a controlled virus challenge experiment was conducted in piglets (Fig. 9A). Piglets received either Verapamil HCl or DMSO prior to a PRRSV SD16 challenge. Blood samples were collected at 3, 7, 10, 14, and 21 dpi for viral load quantification. Subsequently, qPCR analysis revealed significantly lower PRRSV copy numbers in the Verapamil HCl-treated group when compared to the DMSO-treated PRRSV-infected control group (Fig. 9B). The therapeutic efficacy of Verapamil HCl against PRRSV-induced pulmonary damage was also investigated. As shown in Fig. 9C, compared to the DMSO+PRRSV SD16-treated group, the Verapamil HCl-treated group showed minimal pulmonary consolidation. Meanwhile, DMSO-treated PRRSV-infected piglets exhibited thickened alveolar septa and inflammatory cell infiltration, with more extensive lung lesions compared to the Verapamil HCl group. Mock-infected controls showed no detectable pathological lesions (Fig. 9D). Serum IFN-α/β analysis revealed higher IFN production in Verapamil HCl-treated PRRSV-infected piglets than in mock controls (Fig. 9E). Collectively, these *in vivo* findings demonstrated that Verapamil HCl alleviates PRRSV-induced pathological damage in piglets while stimulating IFN-mediated antiviral responses.

## DISCUSSION

Throughout the past three decades, PRRSV has imposed a substantial economic burden on the global swine industry. Despite ongoing research efforts, effective prophylactic vaccines and therapeutic agents for PRRSV control remain limited. The current study demonstrates that Verapamil HCl exhibits significant antiviral activity against diverse PRRSV-1 and PRRSV-2 strains in both PRRSV-susceptible cell culture systems (Fig. 1 and 2) and piglet models (Fig. 9). Our mechanistic investigations revealed that Verapamil HCl effectively attenuates PRRSV-induced Ca^2+^ imbalances (Fig. 4). Additionally, Verapamil HCl inhibits inflammatory responses and enhances IFN-α/β production through p38/Nrf2/Keap-1 signaling pathway modulation (Fig. 5 to 8). These findings provide comprehensive evidence supporting the potential of Verapamil HCl as a novel therapeutic intervention against PRRSV infection, mediated by multiple complementary antiviral mechanisms. Thus, the findings show that Verapamil HCl could hold the key to addressing the current therapeutic gap in PRRSV management strategies (Fig. 3).

Both extracellular and intracellular Ca^2+^, as well as host Ca^2+^ channel proteins, are vital for virus replication and release. Thus, host Ca^2+^ channels act as potential anti-viral targets (17, 37–39). Recent evidence shows that several viruses utilize host Ca^2+^ and Ca^2+^ channel proteins to enable self-replication, thus disturbing Ca^2+^ homeostasis in host cells (20). For instance, Endo et al. reported that SARS-CoV-2 manipulates host Ca^2+^ responses and associated signaling pathways for self-replication (38). Meanwhile, another study showed that some L-type calcium channel blockers (felodipine and nifedipine) inhibit SARS-CoV-2 infection *in vitro* by regulating Ca^2+^ influx (40). Diao et al. found that host Ca^2+^ homeostasis and Ca^2+^ channels play important roles in PRRSV infection (21). Notably, our previous study demonstrated that Diltiazem HCl, an L-type Ca^2+^ channel inhibitor, protects susceptible cells and piglets from PRRSV infection-induced injury by inhibiting the internalization and replication of the virus (20). Meanwhile, the present study showed that Verapamil HCl decreases Ca^2+^ influx in susceptible cells and attenuates PRRSV infection by blocking L-type cellular Ca^2+^ channels (Fig. 4). Unlike Diltiazem HCl, Verapamil HCl exerts anti-PRRSV effects at multiple stages, including adsorption, internalization, replication, and release. Therefore, we speculated that the effect of Verapamil HCl against PRRSV infection may be mediated by mechanisms beyond the blockade of L-type Ca^2+^ channels, warranting further exploration.

The type I IFN system and pro-inflammatory cytokines both play a crucial role in the innate immune defense against pathogens (41, 42). Accumulating evidence shows that PRRSV induces a poor innate immune response during the infection cycle, often leading to decreased IFN production and increased pro-inflammatory cytokine release (43). Some treatment agents exert anti-PRRSV effects by regulating type I IFN and pro-inflammatory cytokine levels *in vivo*. For instance, Wu et al. reported that *Sus scrofa* RNase L inhibits PRRSV replication by promoting type I IFN production and activating related signaling pathways (44). Meanwhile, Hu et al. discovered that Allicin alleviates the pro-inflammatory responses induced by PRRSV to suppress PRRSV replication via MAPK signaling pathway activation (24). Our prior research demonstrated that hyperoside suppresses PRRSV replication by attenuating inflammatory responses and autophagy via the modulation of the TLR4/ NF-κB and p62-Nrf2-Keap1 signaling cascades (35). Meanwhile, the present investigation revealed that Verapamil HCl demonstrates potent anti-PRRSV activity through the suppression of pro-inflammatory mediators (IL-6, IL-8, IL-1β, and TNF-α) (Fig. 5) and enhancement of IFN-α/β production (Fig. 7).

MAPK signaling cascades act as integral components of antiviral immune responses during PRRSV infection (45, 46). The transcriptional regulators Nrf2 and HO-1 serve as pivotal modulators of host antiviral immunity in the context of PRRSV pathogenesis (47). Our experimental data illustrate that Verapamil HCl-mediated anti-PRRSV activity requires Nrf2/HO-1 pathway activation in susceptible cells, and that these effects are concentration-dependent (Fig. 6). Additionally, we observed that Verapamil HCl enhances phosphorylated p38 (p-P38), Nrf2, and HO-1 expression *in vitro* (Fig. 8). These molecular effects are reversed upon treatment with siNrf2, siHO-1, and the p38 antagonist SB203580 (Fig. 6 and 8). These mechanistic findings indicate that Verapamil HCl exerts anti-PRRSV efficacy through p38/Nrf2/HO-1 signaling pathway activation.

**Fig 10:**
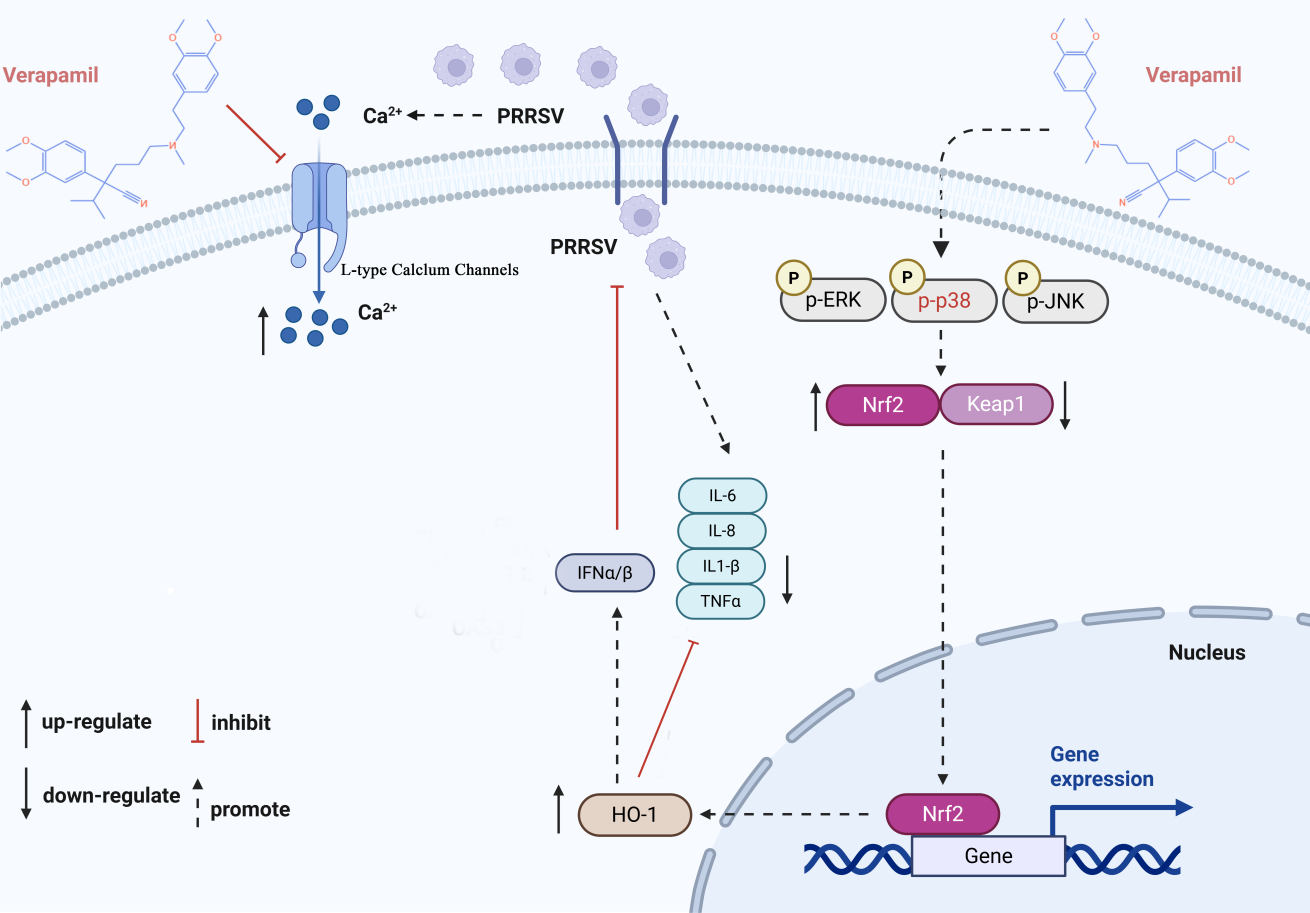
Schematic diagram depicting the anti-PRRSV effects of Verapamil HCl. Verapamil HCl activates AMPK by promoting the expression of p-p38, which activates Nrf2 and its downstream gene *HO-1. HO-1* upregulation promotes the host cellular type I IFN response and inhibits the production of pro-inflammatory factors. Meanwhile, Verapamil HCl reverses the calcium ion imbalance induced by PRRSV. Collectively, these mechanisms inhibit PRRSV infection at multiple stages.

In conclusion, the comprehensive analyses in this study reveal that Verapamil HCl effectively suppresses PRRSV infection both *in vitro* and *in vivo*. On a mechanistic level, Verapamil HCl can reverse the Ca^2+^ ion imbalance induced by PRRSV. In addition, Verapamil HCl causes the p38/Nrf2/HO-1-dependent inhibition of pro-inflammatory factor production and stimulation of IFN responses (Fig. 10). These findings corroborate the therapeutic potential of Verapamil HCl as an innovative antiviral agent for PRRSV control.

## Data Availability

The raw data that support the findings of this study are openly available in ScienceDB at https://doi.org/10.57760/sciencedb.28396 under reference 48.
